# Supplementary material for: On the Risks of Phylogeny-Based Strain Prioritization for Drug Discovery: Streptomyces lunaelactis as a Case Study
Source: Biomolecules. 2020 Jul 10;10(7):1027. doi: 10.3390/biom10071027 (PMC7408125; doi:10.3390/biom10071027)
Supplement: Supplementary file 1 [file biomolecules-10-01027-s001.pdf]

Figure S1

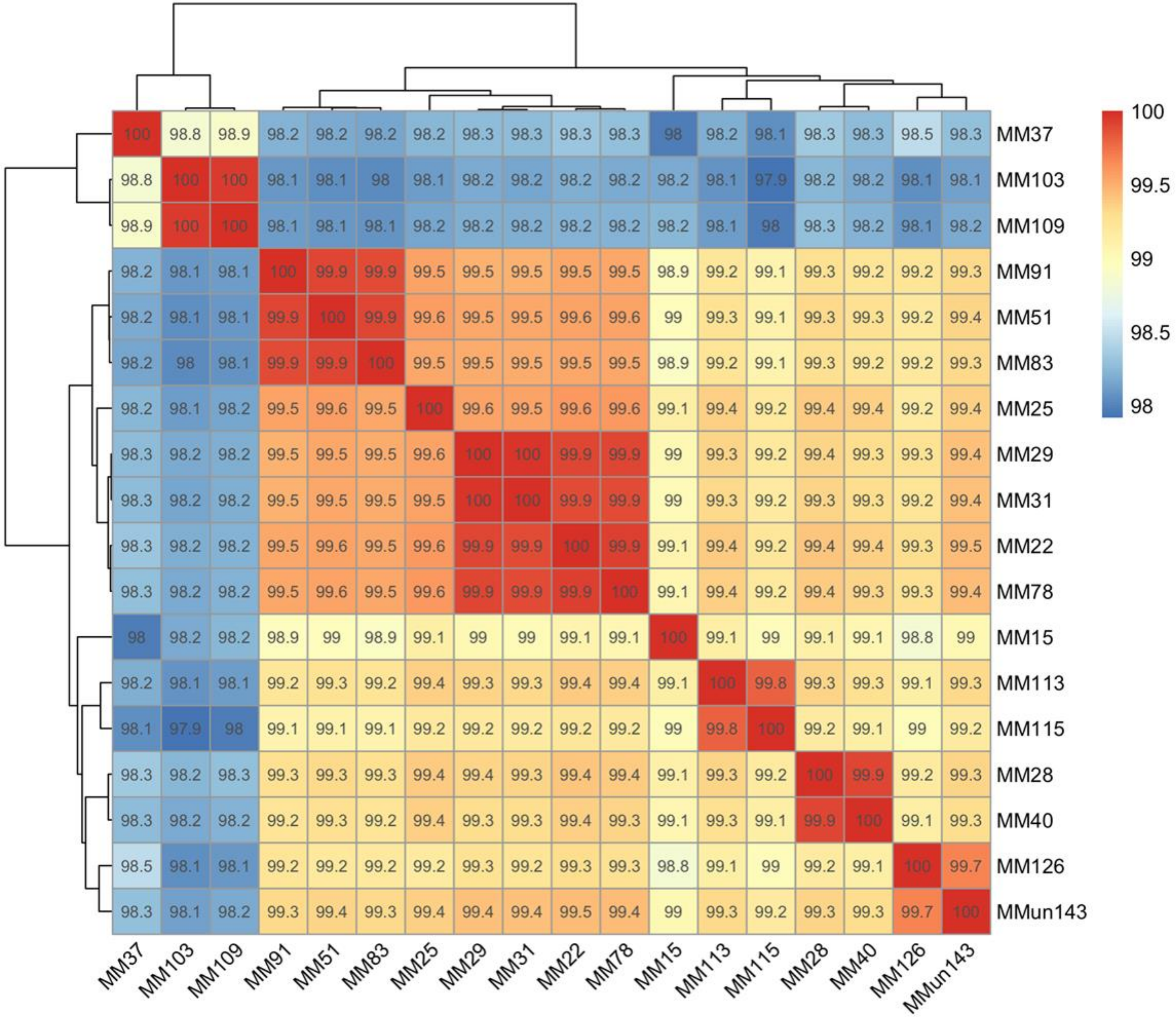

Figure S1. Clustered heatmap showing the average nucleotide identity (ANI) of the 18 *S. lunaelactis* strains genomes.

Figure S2

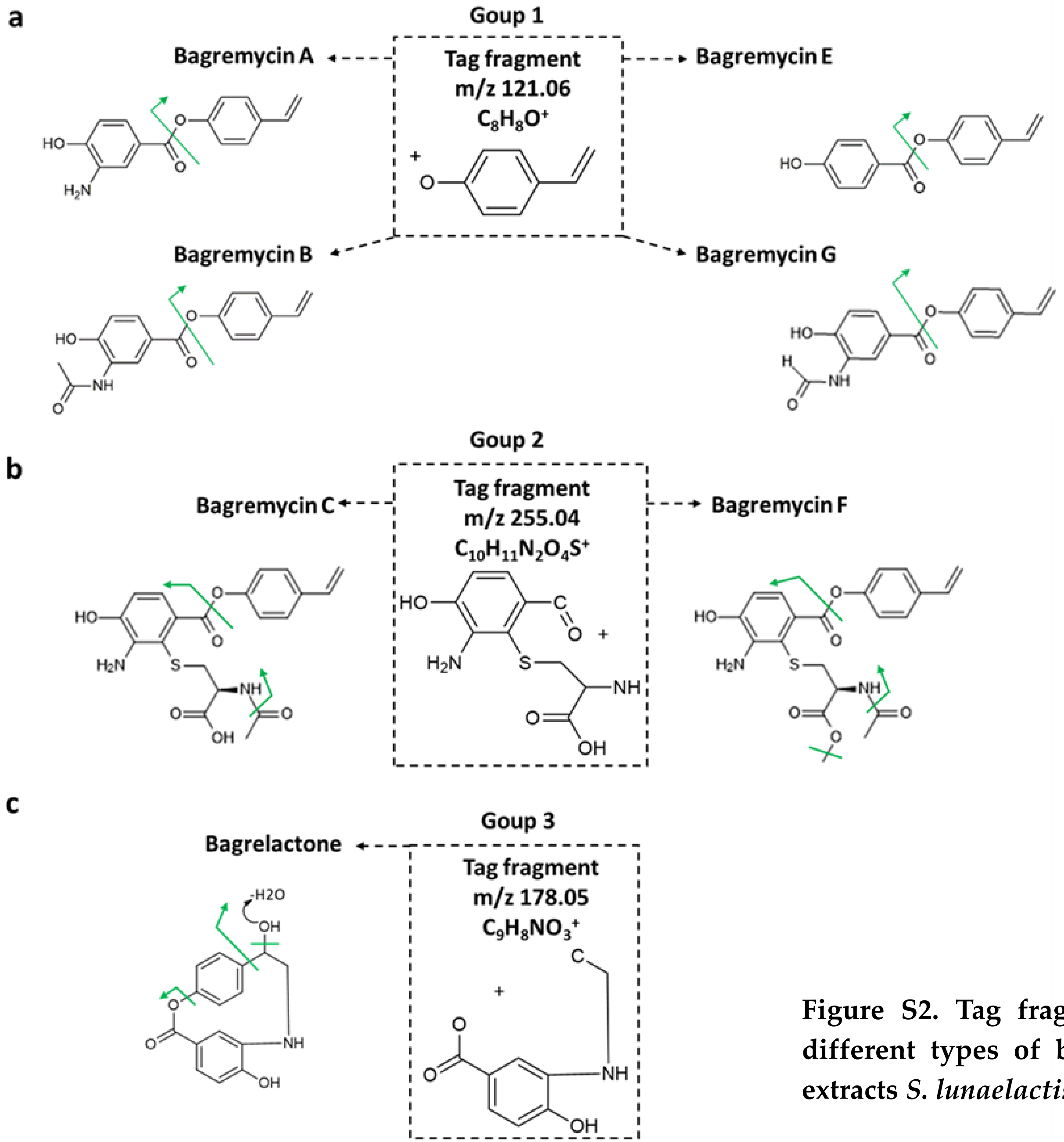

Figure S2. Tag fragments for the identification of the three different types of bagremycin-related compounds in the full extracts *S. lunaelactis* strains.

Figure S3
part 1

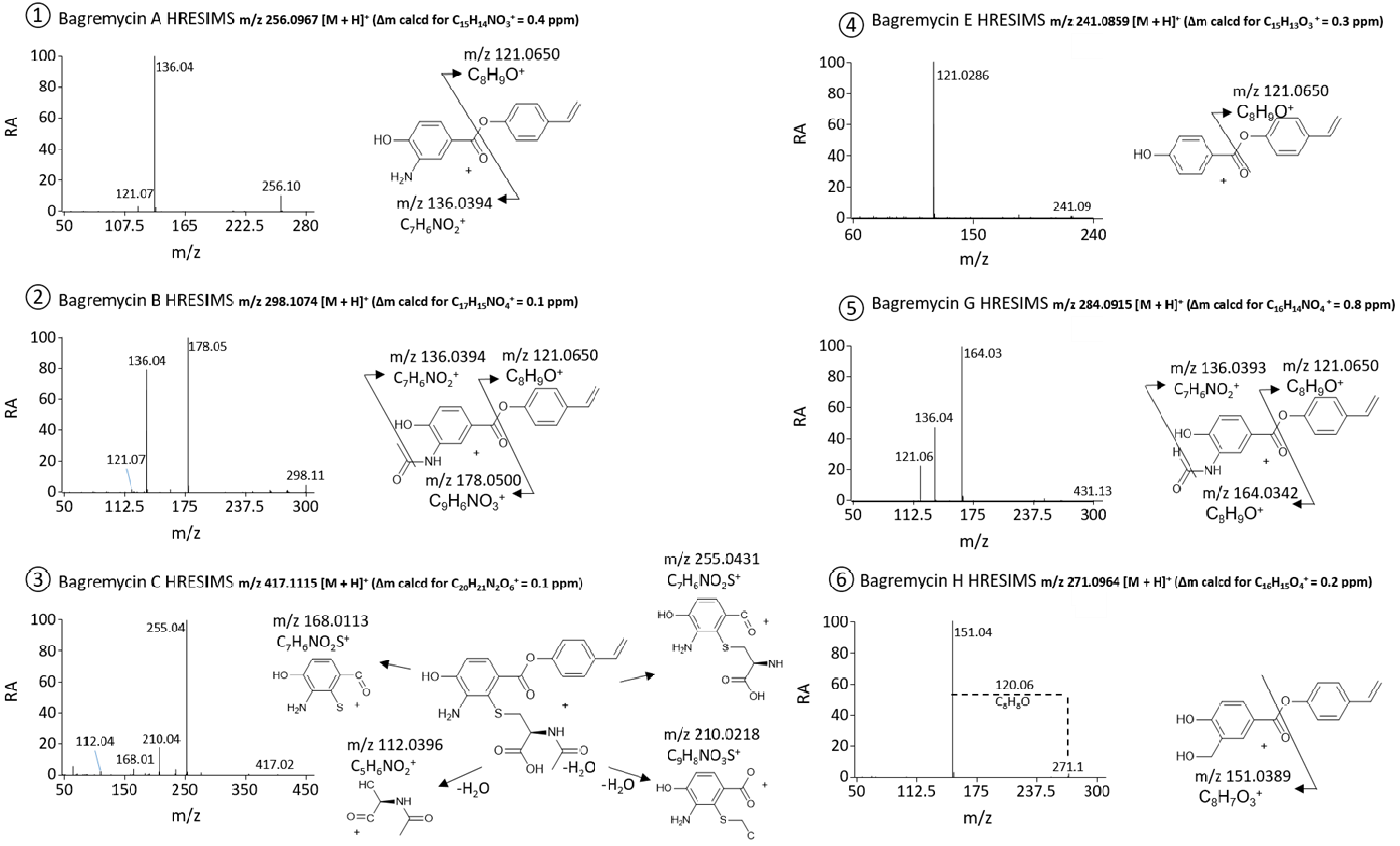

Figure S3
part 2

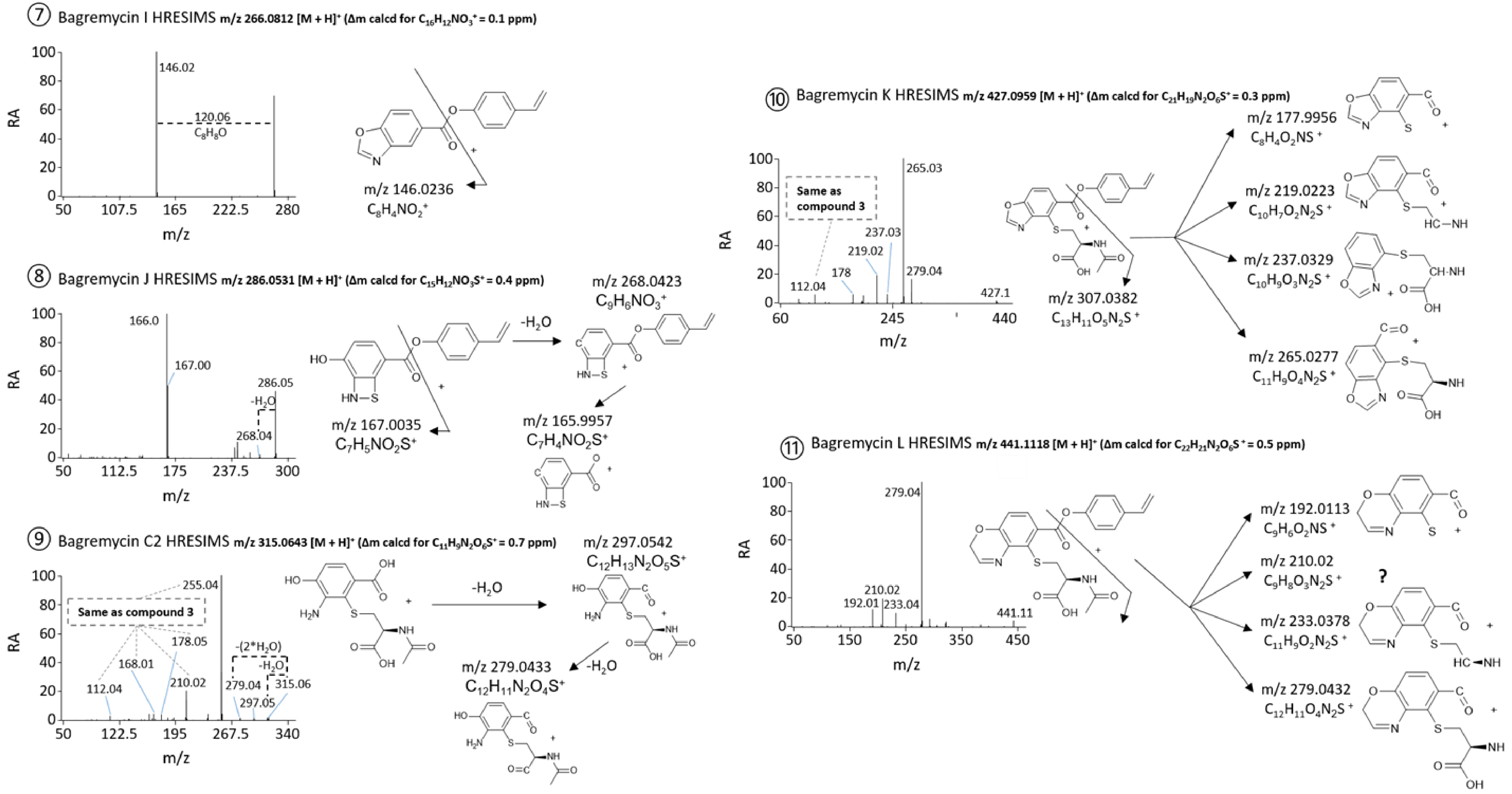

Figure S3. MS/MS spectra of bagremycins identified in the extracts of *S. lunaelactis* strains. The notification "Same as compound x" refers to compounds for which part of their MS/MS spectrum contains the same fragments previously described in the MS/MS spectrum of another compound. A question mark (?) refers to major fragments for which we could not propose a structure.

Figure S4

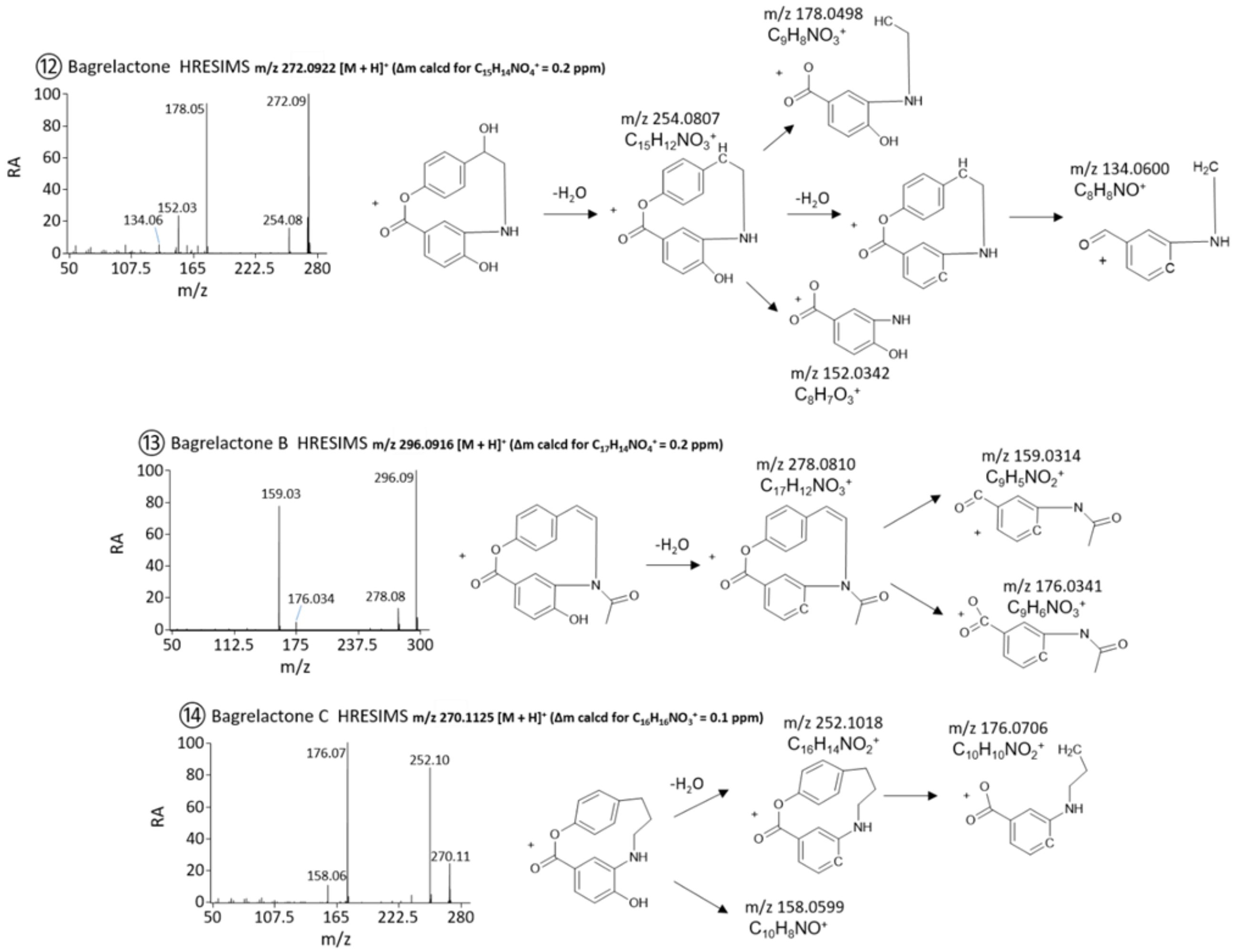

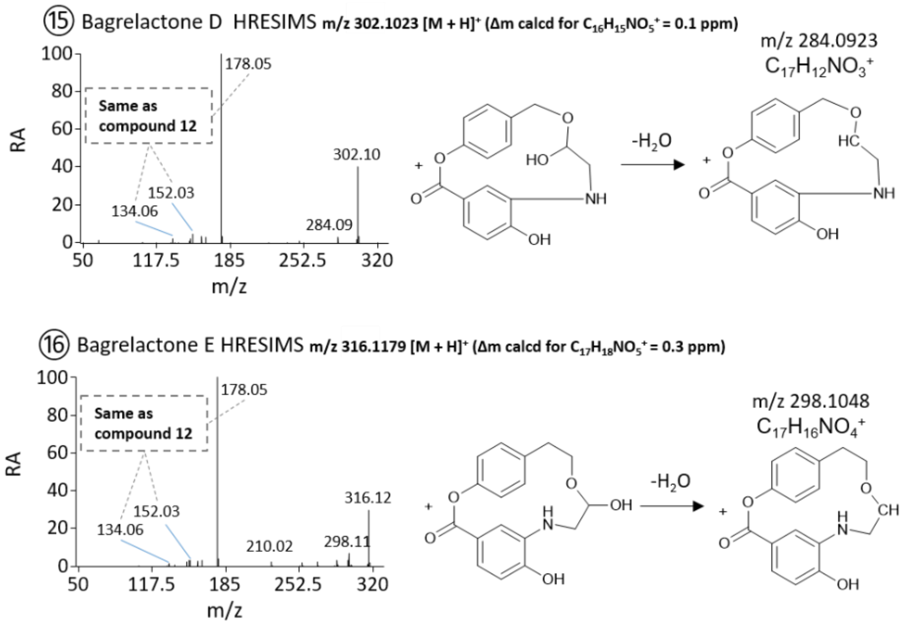

Figure S4. MS/MS spectra of predicted new bagrelactone related compounds identified in the extracts of the various *S. lunaelactis* strains. The notification "Same as compound x" refers to compounds for which part of their MS/MS spectrum contains the same fragments previously described in the MS/MS spectrum of another compound.
